# Response to: Comment on “Serum Amyloid A as a Marker of Persistent Inflammation and an Indicator of Cardiovascular and Renal Involvement in Patients with Rheumatoid Arthritis”

**DOI:** 10.1155/2015/749565

**Published:** 2015-02-23

**Authors:** Bożena Targońska-Stępniak, Maria Majdan

**Affiliations:** Department of Rheumatology and Connective Tissue Diseases, Medical University of Lublin, Ulica Jaczewskiego 8, 20-950 Lublin, Poland

We thank the authors for the detailed analysis of the paper “Serum Amyloid A as a Marker of Persistent Inflammation and an Indicator of Cardiovascular and Renal Involvement in Patients with Rheumatoid Arthritis.” We do appreciate your comments.

However, the roles of some factors influencing serum amyloid A (SAA) concentration, indicated by the authors, have been discussed in the paper—the role of treatment with glucocorticoids and disease modifying drugs (DMARDs), including biological DMARDs [1]. Treatment with nonsteroidal anti-inflammatory drugs (NSAIDs) was not regular; they were taken by the patients on demand, during periods of joint pain exacerbation and in variable doses. That is why reliable analysis of NSAID influence on SAA level was not available. We did not analyze the treatment with statins.

Most of diseases mentioned in the “Letter to the Editor” potentially influencing SAA level did not occur in our patients. The study group consisted of patients with rheumatoid arthritis (RA) and they did not suffer from other types of arthritis (ankylosing spondylitis) or autoinflammatory diseases (familial Mediterranean fever, systemic lupus erythematosus, and vasculitis), inflammatory bowel diseases, acute pancreatitis, psoriasis, epilepsy, and major depression. Diabetes mellitus was found only in 6 patients (4.3%).

Dietary food supplements and antioxidants are not prescribed routinely by physicians and are used by patients themselves. It is also difficult to establish the actual alcohol use. We did not collect the detailed questionnaire of patients' diet and habits; it was not a goal for our study.

However, smoking cigarettes is quite an important factor involved in the pathogenesis and activity of RA. In our study group current smoking was reported by 29 patients (20.7%) and 111 patients (79.3%) did not smoke cigarettes at the time of examination. We analyzed the data and found no correlation between the status of smoking and SAA serum concentration.
